# Supplementary material for: Cytokine Modulation in Breast Cancer Patients Undergoing Radiotherapy: A Revision of the Most Recent Studies
Source: Int J Mol Sci. 2019 Jan 17;20(2):382. doi: 10.3390/ijms20020382 (PMC6359111; doi:10.3390/ijms20020382)
Supplement: Supplementary file 1 [file ijms-20-00382-s001.pdf]

International Journal of *Molecular Sciences*

Table S1. Reasons for study exclusion.

First Author (of Excluded Study)	Exclusion Reason(s) after Analysis	PMID
De Santis F et al. 2018	Reports on the eighth annual conference of "Innovative therapy, monoclonal antibodies, and beyond" held in Milan on Jan. 26, 2018, and hosted by Fondazione IRCCS–Istituto Nazionale dei Tumori (Fondazione IRCCS INT) mainly regarding evidence from preclinical studies in vitro and in vivo	30393044
Masjedi A et al. 2018		30372844
Najafi M et al. 2017	Review where the effects of melatonin on the modulation of immune responses in both normal and tumor tissues are discussed	28510090
Liu LK et al. 2017	Review on the pathogenesis and prevention of radiation-induced myocardial fibrosis for providing references for the prevention and treatment of radiation-induced myocardial fibrosis	28440606
Windrichova J et al. 2017	No RT / treatment indicated: 130 oncological patients with solid tumors (including breast, but no specific indications on these patients' values/results are given), higher levels of MIC1/GDF15 in patients with bone metastases compared with oncological patients without metastases (serum levels assessed by the xMAP technology using Human Cancer Metastases Biomarker Magnetic Bead Panel - Merck Millipore)	28314325
Xiao C et al. 2017	Assessment of plasma cytokines 1 year post-radiotherapy to evaluate possible correlation with fatigue and depressive symptoms	28193310
Mumbrekar KD et al. 2017	Study of possible associations between 22 genetic variants in 18 radio-responsive genes and the risk of developing RT-induced acute skin adverse reactions in a cohort of patients with breast cancer	27816361
Baijer J et al. 2016	Peripheral blood mononuclear cells from healthy normal volunteers	26982083
Mackey JR et al. 2016	No cytokines	26940688
Klionsky DJ et al. 2016	Guidelines for the use and interpretation of assays for monitoring autophagy (3rd edition). NO CYTOKINES	26799652
Recchia F et al. 2015	Patients undergoing immunotherapy	26637906
Yeboa DN and Evans SB. 2016	Review; no reported articles addressed the selected criteria for study inclusion	26617212
Patnaik A et al. 2015	No RT no cytokines	26581242
Fontanella C. 2015	Biphosphonates and denosumab, palliative radiotherapy, bone metastases	26343511
Kladar NV. 2016	Review on effects of the genus *Ganoderma* spp. reporting mainly results of in vitro and in vivo studies; indication on the use of *G. lucidum* in clinical trial as additional therapy to reduce side effects of conventional anticancer treatments	26317382

**Trombetta M. 2015**	Topical application of amnion-derived cellular cytokine solution on irradiated breast skin. No evidence of systemic absorption	26240671
**Golden EB. 2015**	Endpoint: proportion of patients with abscopal response. Safety and survival	26095785
**Jung K. 2015**	Skin toxicity as major side effect of RT for breast cancer. Stimulation with IL-4	26061397
**Jafarzadeh A. 2015**	Chemokine CCL22 may contribute in tumor development. SNP rs223818 may play a role in the susceptibility to breast cancer	25722218
**Bouillet T. 2015**	Benefits from physical activity and sport among cancer patients seem obvious	25660264
**Litchfield K. 2015**	Role of genes involved in the development and progression of TGCT (testicular germ cell tumors)	25609015
**Wang Z. 2014**	IL-8, IL-10, and TNF polymorphisms could serve as useful predictive biomarkers for breast cancer risk among women in East China	25559835
**Markkula A. 2014**	No available data on RT, CHT, RT+CHT, AI, and Tamoxifen treatments	25305747
**Hellen R et al. 2014**	2 cases, no mat and met, no statistical analysis, no cytokine variation	25224250
**Ukaji T and Umezawa K. 2014**	Constitutive expression of NF-kB icancer stem cells	25128192
**Ben Musa R et al. 2014**	1 case, no RT, no quantitative test for cytokines	24914353
**Chien TJ et al. 2014**	Acupuncture and cancer (out of target)	24716183
**Rattay T and Talbot CJ. 2014**	Genetic polymorphism and adverse reaction to radiotherapy	24702740
**Pan K et al. 2014**	Cytokine-induced killer cell immunotherapy (survival study)	24668644
**Whiteside TL. 2014**	Inducible (i) or adaptive Treg in patients with HNSCC and treatments with chemotherapy	24213679
**Chin AR and Wang SE. 2014**	Review on cytokines driving breast cancer stemness and their mechanisms of action	23562748
